# Hair follicle regional specificity in different parts of bay Mongolian horse by histology and transcriptional profiling

**DOI:** 10.1186/s12864-020-07064-1

**Published:** 2020-09-22

**Authors:** Ruoyang Zhao, Wu Yihan, Yiping Zhao, Bei Li, Haige Han, Togtokh Mongke, Tugeqin Bao, Wenxing Wang, Manglai Dugarjaviin, Dongyi Bai

**Affiliations:** 1 lnner Mongolia Key Laboratory of Equine Genetics, Breeding and Reproduction; Scientific Observing and Experimental Station of Equine Genetics, Breeding and Reproduction, Ministry of Agriculture and Rural Affairs; Equine Research Center, College of animal science, Inner Mongolia Agricultural University, Zhao Wu Da Road, Hohhot, 306 010018 Inner Mongolia China; 2 Inner Mongolia Center for Disease Comprehensive Control and Prevention, Hohhot, 010030 China; 3 Inner Mongolia Zhong Yun Horse Industry Group, Xilinhot, 026000 China

**Keywords:** Mongolian horse, Skin, Hair follicle, Histology, Transcriptome

## Abstract

**Background:**

Different morphological structures of hairs having properties like defense and camouflage help animals survive in the wild environment. Horse is one of the rare kinds of animals with complex hair phenotypes in one individual; however, knowledge of horse hair follicle is limited in literature and their molecular basis remains unclear. Therefore, the investigation of horse hair follicle morphogenesis and pigmentogenesis attracts considerable interest.

**Result:**

Histological studies revealed the morphology and pigment synthesis of hair follicles are different in between four different parts (mane, dorsal part, tail, and fetlock) of the bay Mongolian horse. Hair follicle size, density, and cycle are strongly associated with the activity of alkaline phosphatase (ALP). We observed a great difference in gene expression between the mane, tail, and fetlock, which had a greater different gene expression pattern compared with the dorsal part through transcriptomics. The development of the hair follicle in all four parts was related to angiogenesis, stem cells, Wnt, and IGF signaling pathways. Pigmentogenesis-related pathways were involved in their hair follicle pigment synthesis.

**Conclusions:**

Hair follicle morphology and the activity of ALP differ among four body parts in bay Mongolian horse. Hair follicles of the different body parts of the are not synchronized in their cycle stages. GO terms show a regional specificity pattern between different skin parts of the bay Mongolian horse. These results provide an insight into the understanding of the biological mechanism of the hair follicle in other mammals.

## Background

**Fig. 1:**
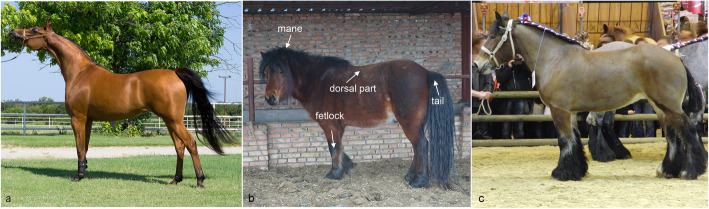
Hair length in different parts of horse showing differences among breeds. **a** is an Arabian horse (https://en.wikipedia.org/wiki/Arabian_horse). **b** is a Mongolian horse; the picture was taken in Inner Mongolia, China. The white arrows point four parts as following: mane, dorsal, tail, and fetlock. **c** is a Trait du Nord, a breed of heavy draft horse (https://en.wikipedia.org/wiki/Trait_du_Nord). **a** and **c** are from Wikimedia Commons under CC-BY-SA copyright, which can be used and adapted

Since about 5500 thousand years ago, horse (*Equus caballus*) has been domesticated as a crucial role in the agricultural industry, transportation, and military activities [1]. Horse hair, with high regional specificity, can be mainly characterized at the mane, dorsal part, tail, and fetlock. Few mammals have such complex hair phenotypes on one individual body; therefore, the horse is a good model for studying hair follicle morphology and development due to the morphological differences existing among breeds and even in the different body parts of the same animal (Fig. 1).

Science and technology have led to the evolution of horse domestication and the production of diversified coat colors [2]. Horse coat color might be changing during the early ages to the adult stage, and also be influenced by environment and nutrition. Compared with young horses, adult horse coat color keeps relatively unchanged. Horse coat color is described as base colors, dilutions, and white spotting and depigmentation patterns [3]. The base color of a horse is generally classified into the black, chestnut, bay, or seal brown and is determined by two interacting loci (extension and agouti) that affect melanocyte function [4, 5]. These two loci determine if eumelanin or phaeomelanin will be produced in their corresponding melanosome. The extension locus (E) encodes the melanocortin-1 receptor (MC1R) and the agouti locus (A) encodes agouti signaling protein (ASIP) which suppresses MC1R. Signaling through MC1R with the agonist results in the production of eumelanin, while antagonist binding (ASIP) results in phaeomelanin production [6]. Bay horse, the most common base color in horses, is the product of the dominant wild type alleles at both loci (A, E). Bay horses have a red body with black mane, tail, ear tips, and lower legs. Over the last 70 years, human and mice hair follicles are extensively studied, and other animal models include pig, cattle, and sheep were also used to study hair follicle biology [7]. Although there are some studies on the horse skin [8, 9], little is on horse hair follicle [10, 11]. Understanding the hair growth, cycle, and melanogenesis in different parts of horse skin provides insights and valuable information for studying the regional specificity of hair follicles in other mammals.

Hair follicle undergoes lifelong cyclical transitions of anagen, catagen, telogen, and exogen (a stage which does not occur at every cycle), which is known as the hair cycle [12]. Through self-renewal and differentiation, stem cells are essential for hair follicle development and cycling, which involve many signaling pathways such as Wnt, Sonic Hedgehog, Notch, and bone morphogenetic protein (BMP). Some growth factors are also involved in the transition from telogen to anagen [13, 14]. Wnt signals are considered to be the most important factors during hair follicle morphogenesis. Wnt signaling pathway facilitates stem cell maintenance and proliferation. Wnt signaling activates the regeneration of hair follicles [15, 16]. Dysregulation of this pathway often results in epithelial cancers [17]. Notch signaling also plays an important role in hair follicle development by maintaining the follicular structure [18, 19]. Notch participates in hair follicle development to control stem cell fate determination [20, 21]. Notch regulates cell differentiation and promotes boundary formation by altering the adhesive properties of keratinocytes [22]. The dermal Wnt pathway upregulates the epithelial Notch expression.

Hair follicle has two mesenchymal components, including dermal papilla (DP) and dermal sheath. The DP is enveloped by keratinocytes at the anagen phase. DP releases insulin-like growth factors that are essential for keratinocyte proliferation and differentiation [23]. Alkaline phosphatase (ALP) is detected in many tissues and organs [24]. ALP activity is also detected in the hair follicle during and after hair development, and this makes ALP a useful marker to indicate the location, shape, and size of the DP in skin specimens [25]. Over the years, scientists have continuously studied the expression pattern of ALP in the specialized structures of hair follicles in various mammals and the detection of ALP expression is often used in developmental studies and clinical trials [26].

Our study aimed to reveal the morphological differences and ALP expression patterns in hair follicles among four different body parts of the Mongolian bay horse. We used transcriptome analysis to show the pathways relevant to hair follicle morphogenesis and pigmentogenesis.

## Results

### Morphological characteristics differ in hair follicles at four body parts

**Fig. 2:**
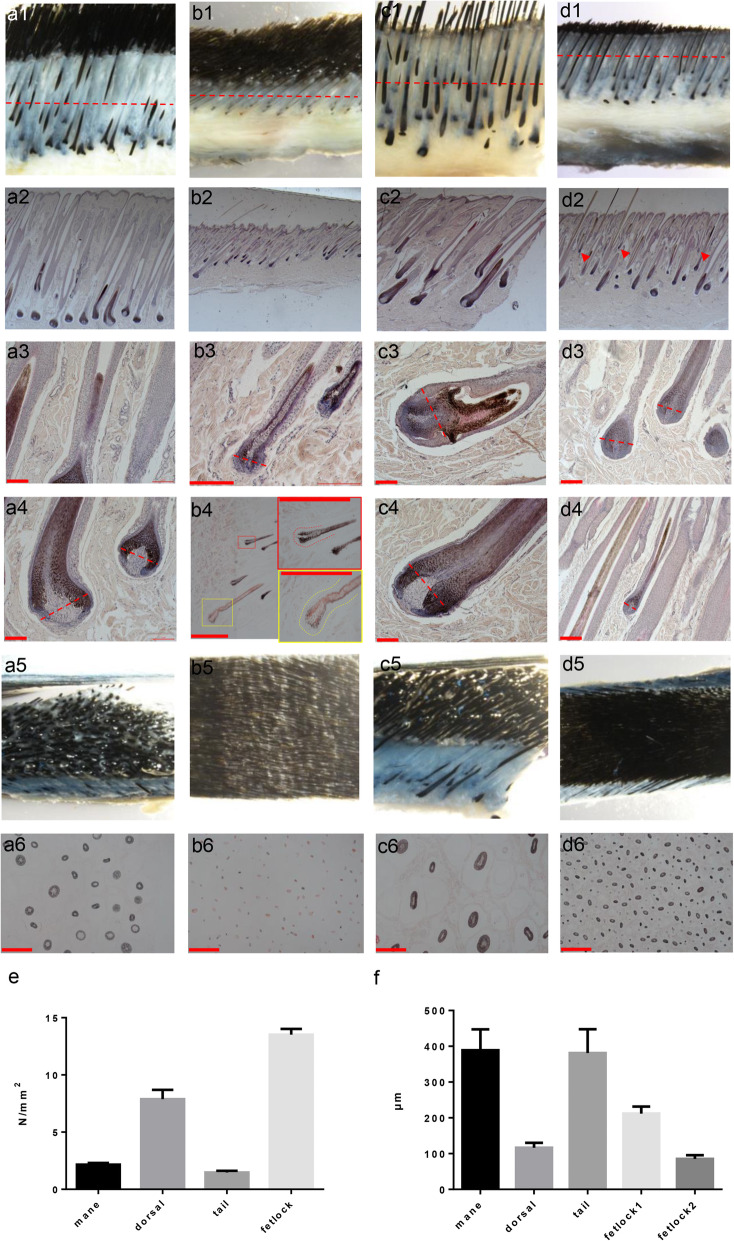
Morphology of Mongolian bay horse hair follicle. From horizontal axis, mane (**a**1–5), body (**b**1–5), tail (**c**1–5), fetlock (**d**1–5); from longitudinal axis, (**a**1-**d**1) and (**a**4-**d**4) are anatomical lens images under 2X amplification. (**a**2-**d**2), (**a**3-**d**3) and (**a**5-**d**5) are microscope images of transparency stain or HE stain. Scale bars are, respectively, 100 μm, 200 μm, and 500 μm, as shown in pictures. **e** is the comparison of hair follicle dermal papilla diameter; **f** is the comparison of hair follicle density

The morphology differed in different parts of hair follicles (Fig. 2 a2, b2, c2, d2). Measurement of hair bulb (HB) diameter of four kinds of hair follicles showed that the tail and mane hair follicles had the biggest HB in diameter (Fig. 2e). The hair density was visibly higher at the dorsal and fetlock regions than that of mane and tail regions (Fig. 2 a6, b6, c6, d6); Statistical analysis showed that hair follicles had the highest density at the fetlock part compared to that of the other body parts (Fig. 2f). The tail hair follicles were highly keratinized compared to the hair follicles at the other body parts (Fig. 2 a3, a4, c3, c4). A cavity was observed in the hair matrix of the tail hair follicle (Fig. 2 c3).

Based on the morphology of human and mouse hair follicles [12, 27], either oval-shaped or onion-shaped DP suggests that the hair follicles in mane, tail, and fetlock regions were at late anagen. In dorsal hair follicles, together with a complete cessation of hair follicle pigmentation and matrix volume loss in the HB, DP was condensed and almond-shaped (Fig. 2 b3), suggesting that most dorsal hair follicles were in early catagen, meanwhile, some follicles remain at late anagen (Fig. 2 b4, amplified in red rectangle). Smaller mid-anagen hair follicles were observed in between the large hair follicles in the fetlock region (Fig. 2 d2 with red arrows, amplified in d4).

Hairs in mane, tail, and fetlock of bay Inner Mongolian horse were observed to be all black, but hairs at the dorsal part include both black and brownish-yellow so that the entire body of the horse showed a brown color (Fig. 2 a1 ~ d1). Paraffin sections with or without H&E staining showed two kinds of pigment granules existed in the dorsal hair follicles (Fig. 2 b4, b6). These included the pheomelanin (amplified in a yellow rectangle), and the eumelanin (amplified in a red rectangle). There was only eumelanin deposition in the hair follicles of the other three parts.

### ALP expression patterns differed in hair follicles among four body parts

**Fig. 3:**
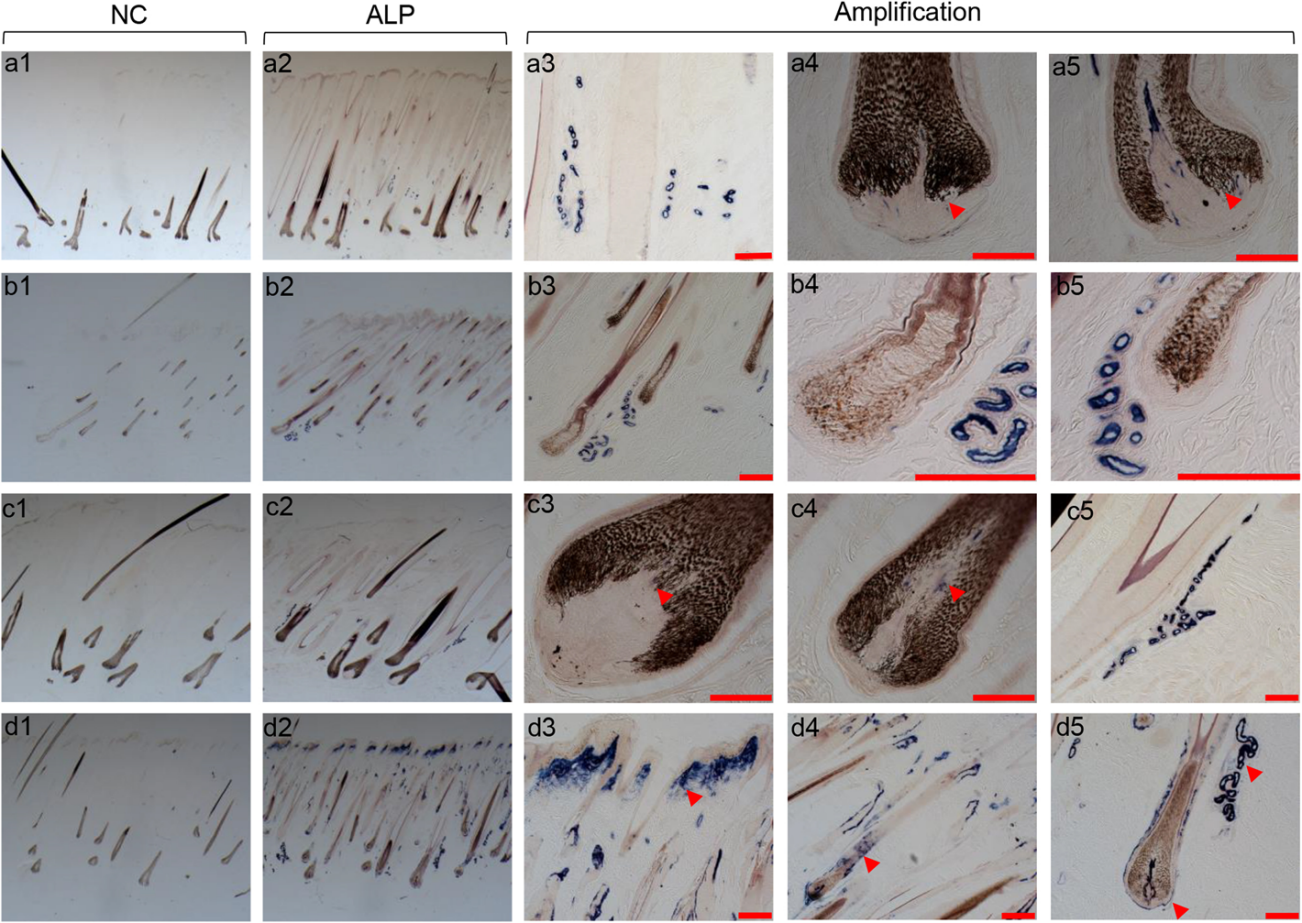
ALP staining results. **a**, **b**, **c** and **d** represent four different parts, respectively, mane, dorsal part, tail and fetlock. Figures a1 ~ d1 are negative control (NC). Figures of a2 ~ d2 are ALP staining. Figures of a3 ~ d5 are the amplifications of ALP staining. a1, a2, × 18; b1, b2, × 40; c1, c2, × 20; d1, d2, × 25; scale bars,100 μm

ALP signals were detected in the vessel-like structures around the hair follicle (Fig. 3 a3, b3, c5, d5) among four body parts. ALP signals were also shown in the DP of mane, tail and fetlock follicles (Fig. 3 a4, a5, c3, c4, d5, red arrowheads), but not found in the dorsal hair follicles (Fig. 3 b4, b5). Different from the others, strong ALP signals were detected in papillary dermis in fetlock skin (Fig. 3 d3, red arrowhead). Meanwhile, ALP signals were also detected in the outer root sheath (ORS) of fetlock hair follicles (Fig. 3 d4, d5, red arrowhead).

### GO terms relevant to hair follicle morphogenesis and pigmentogenesis

**Fig. 4:**
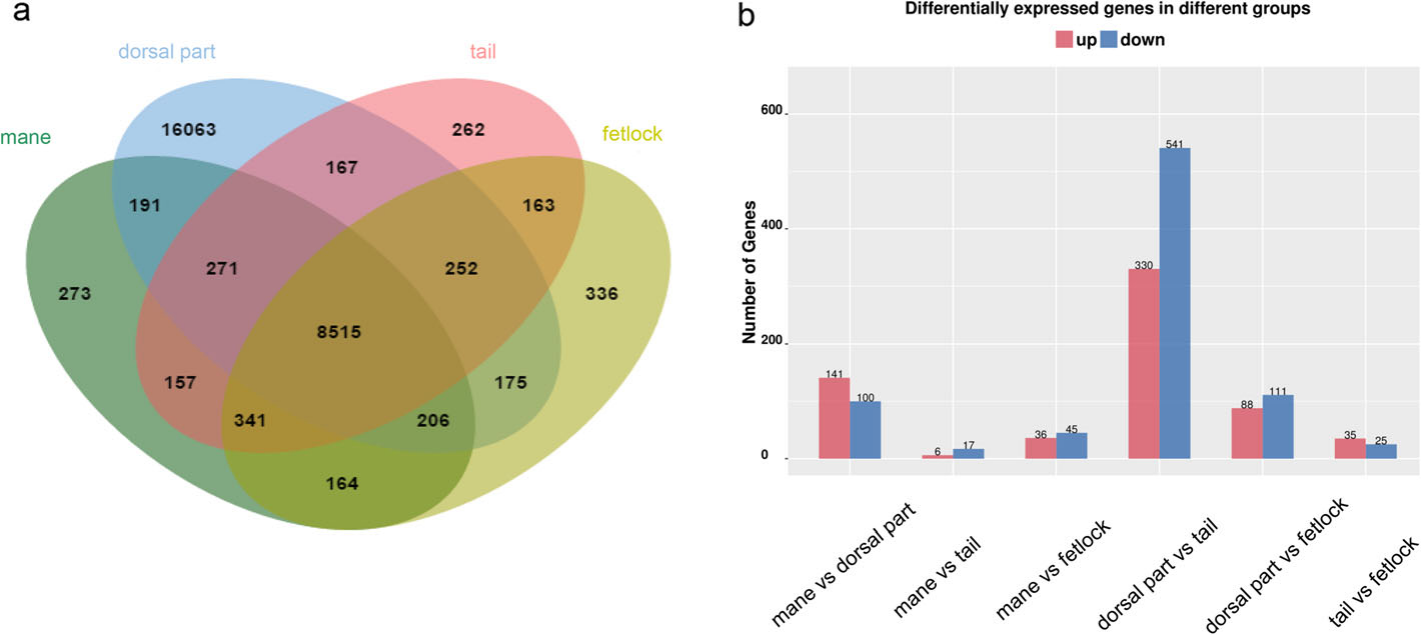
Analysis of differential expression genes. **a** is a Venn diagram of differential expression analysis, different color indicates a different group. Green indicates mane, blue indicates dorsal part, pink indicates tail, and yellow indicates fetlock. **b** indicates the differentially expressed gene numbers between each group, red bars indicate gene numbers which up-regulated, while blue bars indicate down-regulated

A total of 199.28G raw data and 196.49G valid data were obtained by transcriptome sequencing from twelve samples harvested from four different parts of the Inner Mongolian bay horse. Both G20 and G30 met the requirement of the following analysis (Additional file 1). All raw data fastq sequences were deposited at the National Center for Biotechnology Information (http://www.ncbi.nlm.nih.gov/) under BioProject PRJNA477743 with an SRA accession SRP151228. Dorsal skin had the most significant differential gene expression compared to that of skins from the other three parts (*p* < 0.05, Fig. 4a and b, Additional file 2 in detail).

**Fig. 5:**
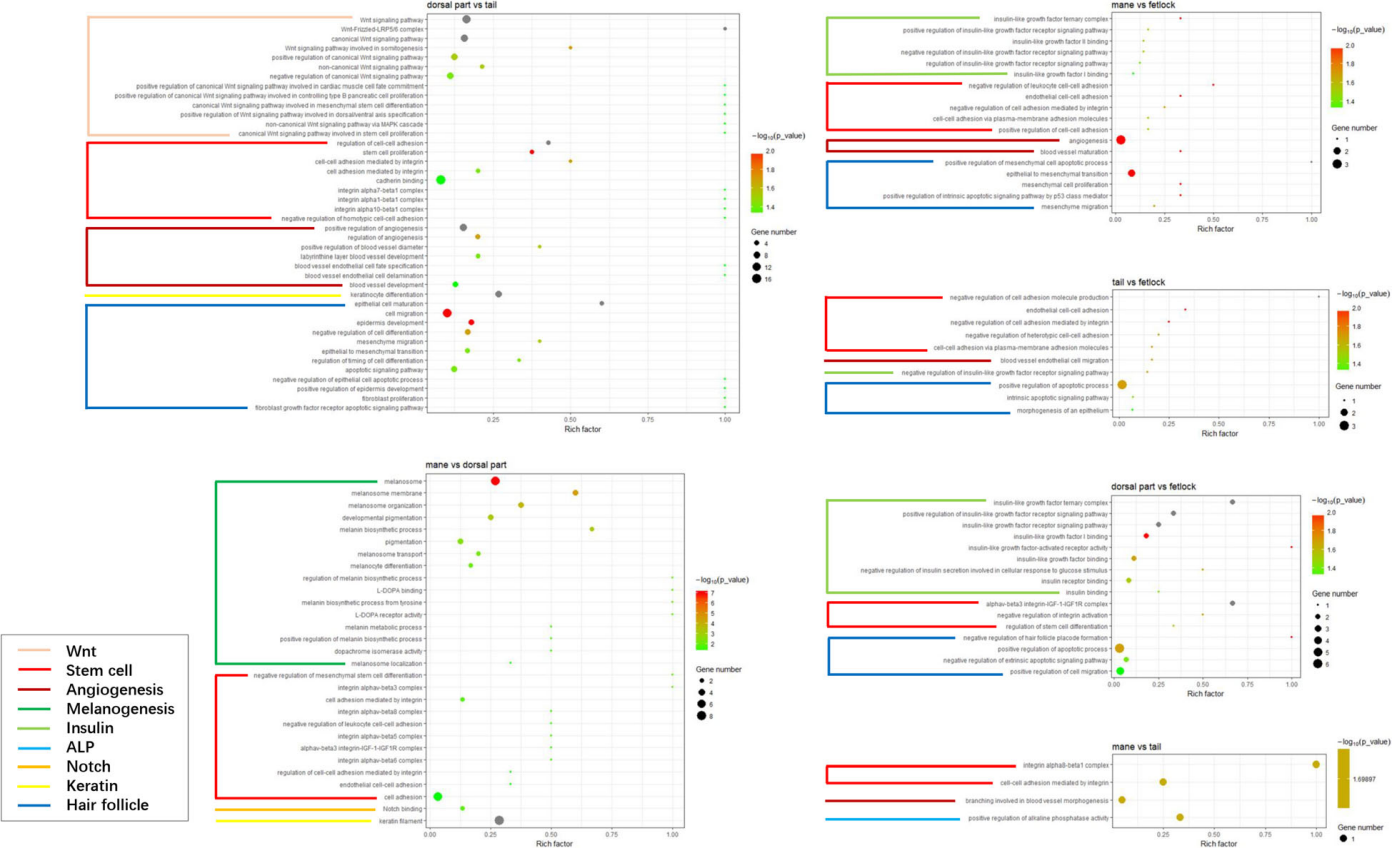
GO enrichment annotated in the Hair Follicle Morphogenesis and Pigmentogenesis related signaling pathway between every two parts. Different color represents different kinds of pathways shown in the bottom left corner; dark blue refers pathways related to hair follicle morphogenesis to some extent but cannot be classified into one accurate function

**Fig. 6:**
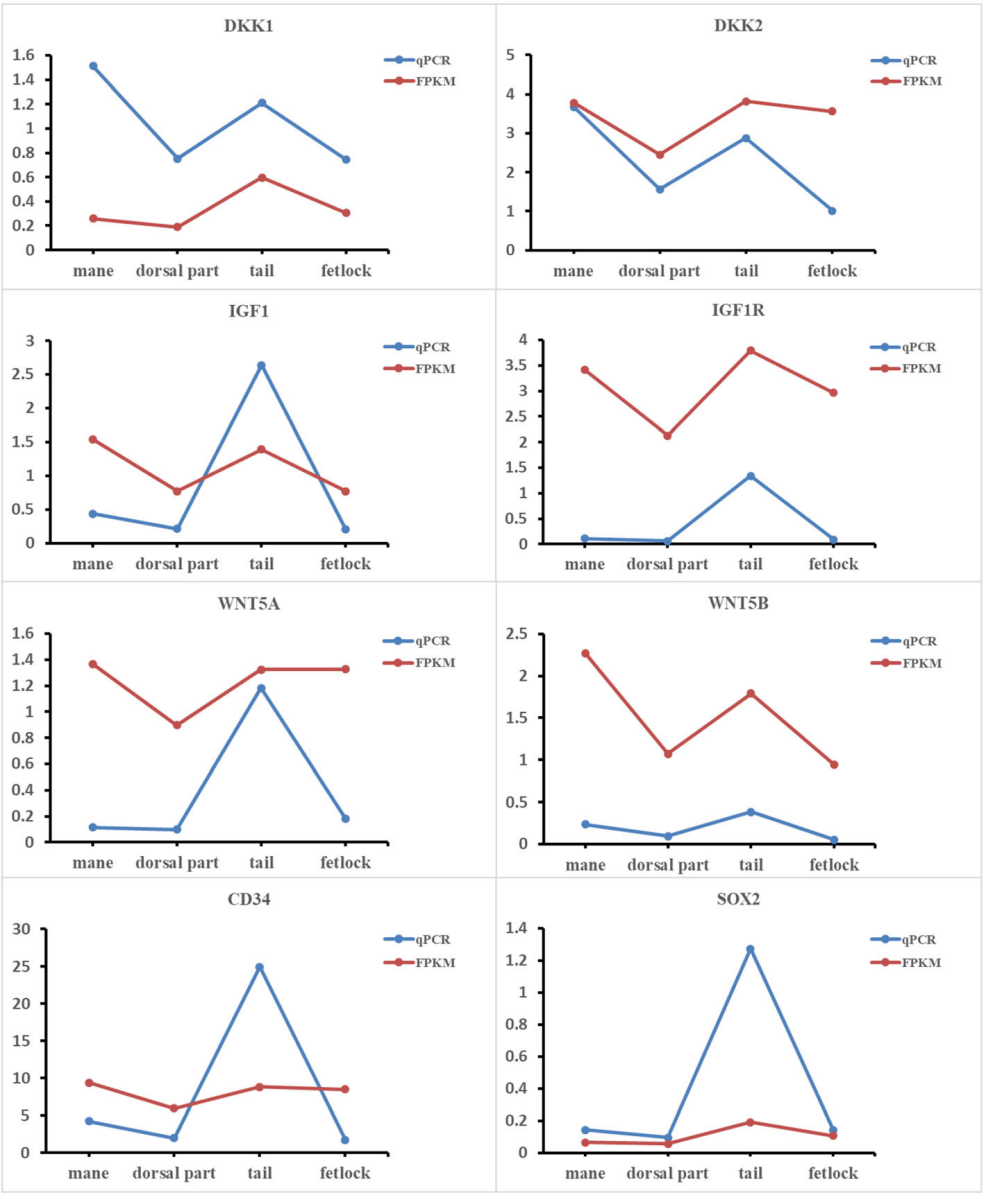
Validation of RNAseq data using qRT-PCR. The red line indicates qPCR results; while blue indicates FPKM values

We selected all GO terms related to hair follicle morphogenesis and pigmentogenesis and found some Intriguingly patterns between every two parts (Fig. 5 and Additional file 3). Wnt, stem cell, and angiogenesis-related signaling pathways were enriched in GO terms with the comparison between the dorsal and tail skin. GO terms were mostly associated with melanogenesis and stem cells between mane and dorsal parts. The majority of GO terms were classified in insulin-like growth factor-related signaling pathways and some stem cell signaling pathways between the dorsal part (or mane) and fetlock. Every two parts of mane, tail, and fetlock were all related to the angiogenesis pathway. An alkaline phosphatase pathway was selected between mane and tail. qRT-PCR was used to validate eight randomly selected genes that showed consistent expression tendency with the RNAseq data (Fig. 6).

## Discussion

Histology of hair follicles in different parts of bay Mongolian horse was revealed for the first time. The results showed that mane and tail hair follicles have a similar morphology. Hair follicles in the dorsal part are very different in pigment, cycle stage, density, and size compared with the other three parts. It is obviously seen that two different kinds of hair follicles exiting in the fetlock skin, which might be considered as primary and secondary hair follicles. The density of hair follicles is very high in the fetlock skin suggesting that the horse needs more hairs to protect their knees. Mammalian hair growth is a dynamic process that depends on proliferation, differentiation, and migration of the matrix cells within the bulb of the follicle, and the hair shape is programmed from the HB [28]. The size of HB determines matrix volume, which may lead to the different hair follicle morphologies in different body parts. In the present study, we proposed that the HB diameter differences may cause complex hair phenotypes in one individual horse.

Hair follicle is a mini-organ that undergoes cyclic growth [12]. Different parts of mammals have different hair follicle cycling duration; hair follicle morphology changes during cyclic growth [27]. ALP activity is widely expressed in actively proliferating or remodeling tissues, as well as in cells with a high metabolic rate [29]. The expression pattern of ALP is slightly different at different hair cycle stages in different animals [26, 30, 31]. It has been reported that the ALP activity can last through the whole hair follicle cycle in mouse dorsal skin. But DP and ORS are only ALP-positive during late anagen and early catagen in rat hair follicles [32]. ALP activity changes dynamically in DP and dermal sheath in mouse vibrissa follicles, it reaches the highest level in the early anagen in DP, decreases by nearly half after the middle anagen, and even less in catagen [31]. ALP activities could also be detected at the proximal DS adjacent to DP, with the highest level at the early anagen [33]. In all, understanding of ALP patterns in horse skin and hair follicle helps to determine the hair cycle. Both H&E staining and ALP expression results showed that mane and tail hair follicles are at late anagen. Normally, horses shed twice a year in spring and fall. Our samples were taken in late fall, a season in the north of China when the hair coat of most mammals becomes thicker. Hair cycle of the animal body might be more closely related to season, the reason why hair follicles in the dorsal skin tend to enter catagen after shedding. Meanwhile, most fetlock follicles remain staying in anagen to produce more hairs to defense the strong northwest wind. Histology shows that the hair follicles of different parts of the horse body are not synchronized in their cycle stages, and the duration of the hair cycle in each of the body parts need further investigation. Due to the limited samples, the existing histological results cannot tell if all the hair follicles in the different four parts going through the same cycle stage simultaneously (such as the first hair cycle in mouse), or they are not synchronized like the hairs on a human scalp. This needs to be addressed in further studies on horse embryo skin in different gestational ages.

The phenotypic differences among the four parts of bay Mongolian horse are significant, also with significant differential gene expression. The enrichment of differential mRNAs and GO items is carried out by comparison between every two parts. In this way, within a certain range of *p*-values, the enriched pathway pattern is preliminarily revealed and summarized. Stem cell relevant pathways are enriched in every two groups suggesting that stem cell signals are needed in the whole process of skin and hair follicle morphogenesis and pigment synthesis. The biggest difference between the dorsal part and tail is the shape of hair follicles, Wnt signals are considered to be mostly involved in its formation. GO terms enriched in melanogenesis is somewhat expected because it is obvious that the hair pigment is the most different phenotype between mane and dorsal part. In our study, an ALP pathway is enriched between the mane and tail, suggesting it can not only be used to determine the cycle stages in different parts of horse hair follicles. In association with the hair cycle, highly regenerative hair follicles are known to express ALP. Regeneration of hair follicles is governed by the reciprocal epithelial-mesenchymal interactions [34]. DP cells are critical to hair growth and regeneration of the HB has regressed in catagen by exhaustion and apoptosis of the bulbar epithelial cells. It has been described that hair inductivity of DP appears to be closely related to ALP activity [33]. Hair follicle is like other organs in need of rich nutrients supplement [35], especially, thick and strong hair follicles like mane and tail. Enough supply of nutrition must be accomplished by abundant capillaries and periodic angiogenesis around hair follicles to satisfy the demands of hair follicle cyclic growth. Therefore, ALP activity could have some other functions in hair follicles more than hair inductivity. Although the arteries and veins are located deep in the dermis, the dendritic capillaries are widely distributed above the bulge area, which provides nutrients, hormones, and immune cells to the skin and also plays an important role in heat transfer. These vascular tissues may affect the process of hair follicle regeneration. This is the reason why alkaline phosphatase, angiogenesis, and stem cell-related pathways are enriched in the relatively thick hair follicles. ALP activity shown in vessel-like structures around hair follicles in different parts might be directly related to angiogenesis and artery morphogenesis. When angiogenesis is inhibited, the initiation of anagen will be delayed [35], which indicates that angiogenesis factors can regulate the activity of hair follicle stem cells and in turn to affect the expression of ALP. The relationship between ALP activity and cyclic hair regeneration and the exact roles of ALP in the horse hair follicles should be addressed by further investigations. In DP cells, a variety of growth factors control the hair follicle cycle, including insulin-like growth factors [36]. GO terms are mostly enriched in IGF pathways between the fetlock and the other three parts. We could propose that IGF relevant pathways are closely related to horse hair cycle and hair density in accordance with histological results. These findings will provide some novel insights in horse hair follicle morphogenesis and melanogenesis.

## Conclusion

Our study unveiled that hair follicles are in different morphology at different body parts of the Mongolian bay horse skin, with significant transcriptome level differences identified by RNA-seq analysis. The hair follicle size, density, and cycle among different types of hair follicles are suggested to be related to ALP activity, angiogenesis, stem cells, WNT, and IGF signaling pathways. These factors and pathways may regulate the regional specificity by controlling the hair follicle morphogenesis and pigmentogenesis. Further studies are required to reveal the hair cycle stages and the molecular mechanism controlling the regional specificity at the different body parts of the Mongolian bay horse.

## Methods

### Sample collection

Three male bay Mongolian horses (10 years old) were used in our experiment (Fig. 1b was one of the horses we used in this study). Four kinds of skin samples from mane, dorsal part, tail, and fetlock were excised from each horse, and a total of twelve samples were taken in December of 2017by trained veterinary technicians. The horses were bought from company (CHINA HORSE INDUSTRY GROUP CO., LIMITED) and raised in the same environment (Hohhot, Inner Mongolia, China). All surgery was performed under combination anesthesia and all efforts were made to minimize horses suffering. Detomidine (0.01–0.02 mg/kg, IV) combined with butorphanol (0.02–0.04 mg/kg, IV) was used for analgesia following standing surgery steps. All horses were still reared carefully after surgery. Samples were collected following the institutional, national, and international guidelines and approved by the Animal Ethical Committee of Inner Mongolia Agricultural University. Half of the samples were put into liquid nitrogen immediately then stored at − 80 °C. The other adjacent half were fixed in paraformaldehyde for 20 h, then were dehydrated with gradient ethanol (70, 80, 80, 100%) and cryopreserved in − 20 °C.

### Histological analysis

Samples immersed in 100% ethanol were embedded into paraffin blocks and cut into sections (Leica RM 2245) of 5-7 μm thickness, after being deparaffinized and rehydrated, then stained with hematoxylin and eosin(H&E), or without any stain (transparency stain), for there were no other colors to disrupt observation of original pigment color) (Fig. 2 b4, a6, b6, c6, d6). We measured the HB diameter of 20 hair follicles under the same magnification from 5 locations from serial longitudinal slides each part and repeated in four parts of skin slides. We chose HBs which were cut right in the middle of hair follicles for measurements. The chosen HBs must be integral and clear structured and the planes we measured the HB diameter are shown in figures (Red dashed lines in Fig. 2 a4, b3, c3 ~ 4, d3). Hair follicle density was measured in 5 different fields using cross-sections and the planes we cut the cross-sections were shown in figures (Red dashed lines in Fig. 2 a1, b1, c1, d1). The method above was applied to a total of 12 samples. Data were made graphics using GraphPad (San Diego, CA, USA).

### Histochemistry

Steps of alkaline phosphatase activity detection were as follows: the NBT/BCIP (Maixing, Fuzhou, China) was added on the tissues after gradient alcohol rehydration in the dark for 15 min until a dark blue signal appeared and was stopped by washing with PBS (pH 7.2 ~ 7.4). This was applied to the 12 samples. Signals were detected and measured through digital photographs using the microscope imaging system (Axio Observer D1, ZEISS).

### Library construction and sequencing

Total RNA were extracted from twelve samples (three replicates from each of four parts) stored in − 80 °C in accordance with standard procedure of Trizol reagent (Invitrogen, CA, USA) and Animal Tissue RNA Purification Kit (LC Science, Houston, TX). The quantity and purity of RNA (RIN number > 7.0) were analyzed according to the previous research [37]. After, a certain amount of RNA was purified [37]. Following, the RNA fractions were fragmented by cations under suitable temperature. Finally, cDNA library was constructed using the stranded procedures of mRNA-seq Library Prep Kit (Illumina, San Diego, USA). An Illumina Hiseq 4000 platform was then performed the sequencing [37].

### Transcripts assembly

Cutadapt [38] was used to remove the low-quality fragments. There were two steps we used to wipe out low-quality reads to obtain clean reads. Firstly, we wiped out low reads with adapters and poly-N (N indicates that the base cannot be confirmed) over the rate of 10% using Cutadapt. Secondly, we wiped out paired reads with low-quality through single-ended sequencing (With low-quality bases over the rate of 50% of the whole reads were considered to be the low-quality paired reads). Then, Q20 and Q30 were calculated using q30.py (https://github.com/d-ayedepps/q30). FastQC [37] was used to verify the sequence quality. High-quality clean data obtained were used to do mapping of *Equus caballus* genome (http://asia.ensembl.org/Equus_caballus/Info /Index) by Bowtie2 [39] and TopHat [40]. Reads mapped to the genome were assembled using StringTie [41] and generated a GTF file for each sample. Cuffmerge merges the GTF files into more comprehensive transcripts annotation results. Then, we used Cuffdiff to do gene expression difference analysis [42].

### Expression analysis of mRNAs and GO enrichments

StringTie [41] was used to perform expression level analysis for mRNAs by calculating FPKM [43]. The fragments per kilobase of transcript per million (FPKM) mapped reads was calculated for each gene based on the length of the gene and reads counts mapped to this gene. FPKM simultaneously considers the effect of sequencing depth and gene length for the reads count. The differentially expressed mRNAs were selected with |log_2_^(fold change)^ | < 1 and with statistical significance (*p*-value < 0.05) by R package Ballgown [44]. The Benjamin-Hochberg method was used to correct the *P*-value. GOseq R package was used to selected Gene Ontology (GO) enrichment of differentially expressed genes [45] with a *P*-value of < 0.05.

### Quantitative real-time PCR (qRT-PCR) validation

RNA was used to perform a reverse transcription PCR using HiScript® II qRT SuperMix for qPCR (Vazyme). Subsequently, qPCR was performed using the primers (Additional file 4) from Invitrogen with SYBR®Premix Ex Taq™ II (TaKaRa). A total of 8 genes (*DKK1*, *DKK2*, *IGF1*, *IGF1R*, *WNT5A*, *WNT5B*, *CD34*, and *SOX2*) were randomly selected for qRT-PCR validation. Each sample has 3 replicates. GAPDH as the housekeeping gene was used to assess the level of RNA expression. Data collection and analysis were performed respectively by CFX96 Real-Time PCR Detection System (Bio-Rad) and Microsoft Excel.

## Supplementary information

**Additional file 1.** The quality control of RNAseq data.

**Additional file 2.** Gene differential expression results. Significant gene differential expression results between every two different parts.

**Additional file 3.** GO enrichments. Significant GO enrichments between every two different parts.

**Additional file 4.** Primers for qRT-PCR validation.

## Data Availability

All raw data fastq sequences are deposited at the National Center for Biotechnology Information (http://www.ncbi.nlm.nih.gov/) under BioProject PRJNA477743 with SRA accession SRP151228. All raw sequences are deposited as BioSamples SAMN09478216, SAMN09478217, SAMN09478218, SAMN09478219, SAMN09478220, SAMN09478221, SAMN09478222, SAMN09478223, SAMN09478224, SAMN09478225, SAMN09478226 and SAMN09478227 (https://www.ncbi.nlm.nih.gov/sra/ PRJNA477743). *Equus caballus* genome (http://asia.ensembl.org/Equus_caballus/Info /Index) was used to do mapping.

## Abbreviations

DEGs: Differentially expressed genes
FDR: False discovery rate
FPKM: Fragments per kilobase of transcript per million fragments mapped
GO: Gene ontology
HE: Haematoxylin-eosin
PCA: Principal component analysis
q-PCR: Quantitative real-time PCR
RNA-Seq: RNA sequencing
